# Frequency Domain-Based Super Resolution Using Two-Dimensional Structure Consistency for Ultra-High-Resolution Display

**DOI:** 10.3390/jimaging10110266

**Published:** 2024-10-23

**Authors:** Yu Lim Seo, Suk-Ju Kang, Yeon-Kug Moon

**Affiliations:** 1 Samsung Electronics, Suwon-si 16677, Gyeonggi-do, Republic of Korea; yulim.seo@samsung.com; 2 Department of Electronic Engineering, Sogang University, Seoul 04107, Republic of Korea; 3 Department of Artificial Intelligence and Data Science, Sejong University, Seoul 05006, Republic of Korea

**Keywords:** interpolation, image up-scaling, super resolution, deep learning

## Abstract

Recent advancements in the field of super resolution (SR) have seen the adoption of generative adversarial networks (GANs) for realistic images. In this case, when performing with low-resolution (LR) images, several challenges arise due to the loss of high-frequency details from high-resolution (HR) images, potentially leading to unwanted distortions in the generated SR images. Our paper presents a novel solution by using two-dimensional structure consistency (TSC) for image analysis. The TSC serves as a mask, enabling adaptive analysis based on the unique frequency characteristics of different image regions. Furthermore, a mutual loss mechanism, which dynamically adjusts the training process based on the results filtered by the TSC-based mask, is introduced. Additionally, the TSC loss is proposed to enhance our model capacity to generate precise TSC in high-frequency regions. As a result, our method effectively reduces distortions in high-frequency areas while preserving clarity in regions containing low-frequency components. Our method outperforms other SR techniques, demonstrating superior results in both qualitative and quantitative evaluations. Quantitative measurements, including PSNR, SSIM, and the perceptual metric LPIPS, show comparable PSNR and SSIM values, while the perceptual SR quality is notably improved according to the LPIPS metric.

## 1. Introduction

**Figure 1:**
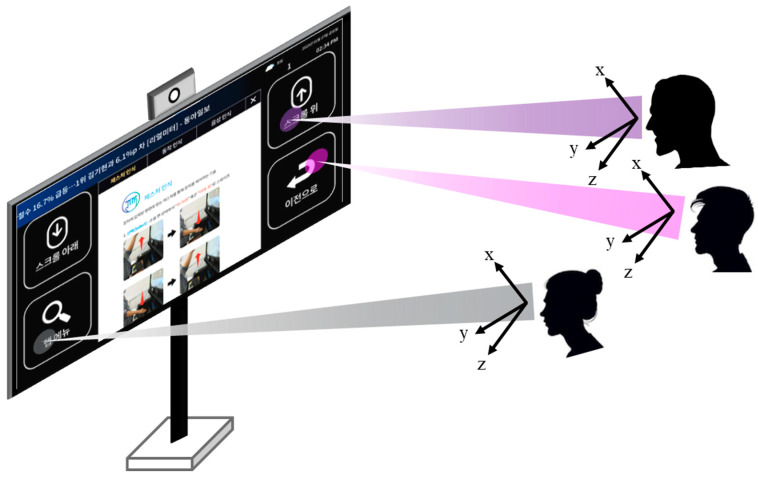
An example of gaze tracking on a high-resolution large display.

With the recent increase in display sizes and growing demand for immersive displays, there is a rising need for ultra-high-resolution images. For instance, as shown in Figure 1, various interfaces such as gaze tracking are being applied to large displays. In such cases, high-resolution images tailored for interfaces are essential for accurate position estimation. Therefore, there is a substantial demand for the output of high-resolution (HR) displays for existing low-resolution (LR) images. Super resolution (SR) is a long-standing challenge in the field of computer vision, involving the enhancement of HR images from their LR counterparts. Recently, many approaches in the field of deep learning-driven super resolution techniques have prominently utilized the adaptability of convolutional neural networks (CNNs), outperforming traditional methodologies [1,2,3]. These methods analyzed various features by using rule-based feature extraction techniques to account for image characteristics, thereby enhancing resolution according to the specific features. However, the performance is limited, as the effectiveness of rule-based methods can vary depending on the feature extraction techniques employed. In order to solve this problem, deep learning-based SR approaches have been proposed to minimize the pixel-wise mean square error (MSE) between HR and the subsequently generated super-resolved images [4,5,6,7]. Training is performed towards reducing pixel-wise discrepancies, yielding elevated peak signal-to-noise ratio (PSNR) and structure similarity index (SSIM) metrics. Nevertheless, these metrics frequently diverge from human perceptual quality standards, thereby imposing constraints to achieve superior image quality across various cases. Hence, Ledig et al. [8] introduced generative adversarial networks (GANs) [9] to the field of single-image super-resolution (SISR), successfully reconstructing perceptually impressive images compared to traditional MSE-based methods. Their approaches leveraged adversarial learning and the concept of the perceptual loss [10]. Several other methods, such as EnhanceNet [11], ESRGAN [12], SROBB [13], and SPSR [14], adopted GANs to produce more photorealistic images in SISR tasks. However, when utilizing GANs with perceptual loss to generate HR images, a notable drawback has been the emergence of undesired distortions in certain regions, deviating from the original HR image. Mechrez et al. [15] integrated the contextual loss [16] into the network proposed by Ledig et al. [8] to preserve the natural characteristics of images. The contextual loss maintains the quality of the image restoration by considering the distribution of mid-level features from a pre-trained image classification network like VGG [17] and aligning the spatial distribution of pixels between HR and SR images. Although training with the contextual loss reduces structural distortion, it can lead to the loss of fine details in some regions, resulting in blur artifacts. To utilize the benefits of both contextual loss and perceptual loss while mitigating their respective drawbacks, the novel concept of mutual loss is introduced. This loss function allows us to adaptively control the training process by identifying image characteristics in the frequency domain, offering a promising solution to improve SISR outcomes. To control the training process, our approach places a deliberate emphasis on the retrieval of missing high-frequency components during the LR image generation for training.

Typically, the creation of LR–HR paired training datasets employs an ideal downsampling filter. While reducing the image to a lower resolution, it inevitably discards high-frequency components and retains a limited amount of low-frequency information. However, high-frequency elements, such as edges and lines, hold substantial significance in assessing perceptual SR performance, and hence, it is required to effectively restore these components. Recently, several methods have been introduced to enhance perceptual SR performance by delving into image analysis within the frequency domain. For instance, Deng [18] implemented a division of the generated image into two separate domains for PSNR-oriented SR and perceptual-based SR, focusing on low- and high-frequency components, respectively. This division was facilitated through the use of 2D stationary wavelet transform and culminated in the fusion of the two domains after image quality enhancement. However, this method has performance limitations because it distinguishes by frequency through a simple application of wavelet transform. In addition, Fritsche [19] adopted a technique of frequency separation to exclusively leverage GAN learning in regions rich with high-frequency components, while relying on a pixel-wise loss function for regions dominated by low-frequency data. This dual-approach method effectively uses the network to restore high-frequency components not presented in the LR image. However, this method has performance limitations because it does not take the structure of the image into account when distinguishing by frequency.

Therefore, our approach uses adaptive training, leveraging the two-dimensional structure consistency (TSC) to differentiate between high- and low-frequency components in the HR image. This TSC acts as a dynamic mask, enabling the network to assess each frequency region and adjust the balance between perceptual and contextual losses. In the low-frequency domain, the preservation of image details is prioritized by enhancing the perceptual loss through mask adjustments. Conversely, in the high-frequency domain, the contextual loss is emphasized to prevent structural distortions or noise artifacts. Additionally, the novel TSC loss is introduced to enhance the accuracy of our TSC by reducing discrepancies between the two-dimensional structure consistencies of the generated SR image and the original HR image. In summary, our main contributions are as follows.

- The structure consistency concept, for the first time, in a GAN-based super resolution network is proposed. It enables our network to effectively restore missing high-frequency components in the HR image, enhancing the network’s capacity to comprehend HR image characteristics within the frequency domain.
- The TSC is proposed as a guiding principle for the in-depth analysis of HR image characteristics in the frequency domain. Subsequently, a novel mutual loss is introduced to utilize a TSC analysis-based mask. This innovative approach allows us to use the benefits of both perceptual and contextual losses while mitigating their respective limitations.

## 2. Proposed Method

To enhance the image quality, the loss function used for training a generator (*G*) typically revolves around minimizing the pixel-wise L1 distance between HR and SR images as follows.

(1) $$L_{px}^{G}=E{\left\|I^{HR}-G\left(I^{LR}\right)\right\|}_{1},$$

where *I^LR^* and *I^HR^* are input LR and HR images, respectively. While this approach may be straightforward in formulation and exhibit a relatively strong SR performance, it faces a significant drawback. As previously noted, training solely based on minimizing pixel-level errors leads to the undesirable outcome of restoring blurry images. Techniques relying on pixel-wise loss, exemplified by models like EDSR [20] and RCAN [21], demonstrate high PSNR and SSIM values but often suffer from suboptimal perceptual quality and insufficient detailing. Moreover, GAN-based networks employ adversarial loss [9] as a key component in their loss function. This approach minimizes the distinction between a generated (fake) image and an authentic (real) image by instructing the discriminator (*D*) to discern between two images. In this context, the fake image corresponds to the one restored by the generator, while the real image is the HR counterpart. The formulation of the adversarial loss for the generator is expressed as follows.

(2) $$L_{a}^{G}=-E\left[\log D(G(I^{lR}))\right].$$

This approach exhibits superior perceptual qualities compared to methods relying on pixel-wise loss, leading to the generation of more visually realistic images. In our training method, the two loss functions are used alongside our novel contributions: the mutual loss and the TSC loss. The subsequent subsections detail the formulation and significance of our two proposed loss functions.

### 2.1. Mutual Loss

Perceptual loss, an important component in leading perceptual-driven SISR methods like SRGAN [8] and ESRGAN [12], is centered on minimizing the gap between the output features of HR and SR images within the VGG network [17]. In contrast, contextual loss not only considers the distance but also the distribution of these output features, thereby ensuring that the restored SR image aligns more closely with the HR image. These two loss functions play a crucial role in achieving the restoration of images with high perceptual quality. Consequently, GAN-based single image SR methods integrate these functions through adversarial learning. Furthermore, in our approach, the characteristics of the HR image are used during network training. Contextual and perceptual losses are dynamically used, thereby leveraging their strengths while mitigating potential drawbacks. As mentioned earlier, the perceptual loss minimizes the L1 distance between the output features extracted by the VGG network. Typically, the perceptual loss leverages high-level features from a pre-trained VGG network during training. This approach aids the super-resolution decoder in capturing intricate details, culminating in a highly perceptual SR performance. Nonetheless, the resulting images often exhibit unwarranted structural distortions, particularly in high-frequency regions characterized by numerous edges and texture components. In contrast, the contextual loss places emphasis on the distribution of output features. This focus contributes to restoring images that closely resemble HR counterparts, mitigating structural distortions and minimizing noise in the process. Hence, our mutual loss is used to enhance the restoration of missing high-frequency components in high-frequency regions, addressing concerns about undesirable structural distortions while simultaneously preserving image details in low-frequency regions. To implement the mutual loss based on the frequency components of the image, a binary segmentation mask derived from a TSC of the HR image is extracted.

**Figure 2:**
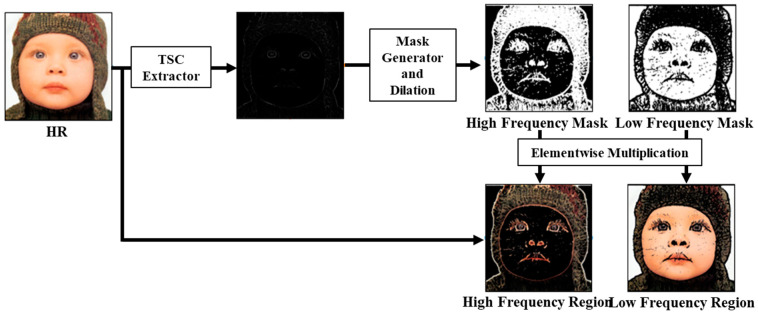
Approach for generating a binary segmentation mask based on the TSC extracted from the HR Image. The HR image comprises low-frequency and high-frequency components that require separate restoration. To address this, the TSC-derived HR mask is used to implement an appropriate training method for each frequency region during the optimization of the network.

Figure 2 illustrates the creation of the TSC-based HR mask. The HR mask for the high-frequency region (*M_h_^HR^*) assigns a value of 1 to areas with TSC values exceeding a predetermined threshold (*th_h_*), signifying the high-frequency region, and a value of 0 elsewhere, representing the low-frequency region. This mask is then applied elementwise to the training image, effectively partitioning the image into adaptive regions. Conversely, for the low-frequency region, the output mask (*M_l_^HR^*) is generated in contrast to the high-frequency mask. It assigns a value of 1 if the TSC value is below *th_l_* and 0 if it exceeds this threshold.

Subsequently, the mask for the high-frequency region retains the input for the contextual loss, rendering the remainder as a black image to exclude it from contextual loss training. Conversely, in the low-frequency region, the input for the perceptual loss is retained, while the remaining region is transformed into black, eliminating any contribution to the perceptual loss. To enhance the boundary representation, the dilation method is used with a disk size of 3. Consequently, the proposed mutual loss enhances SR performance by dynamically applying the training approach based on the frequency components of an image. The formulation of the proposed mutual loss is as follows.

(3) $$L_{M}^{G}=\alpha \cdot L_{P}\left(I^{SR}{}^{\circ}M_{l}^{HR},\;I^{HR}{}^{\circ}M_{l}^{HR}\right)+\beta \;\cdot L_{CX}\left(I^{SR}{}^{\circ}M_{h}^{HR},\;I^{HR}{}^{\circ}M_{h}^{HR}\right),$$

where α and β are the trade-off coefficients of the perceptual loss (*L_P_*) and contextual loss (*L_CX_*) terms, respectively. In addition, ° denotes the element-wise multiplication.

### 2.2. Two-Dimensional Structure Consistency Loss

The primary goal of incorporating the TSC loss is to recover absent frequency components during the generation of a SR image from a LR counterpart. To stimulate the restoration of high-frequency elements not presented in the original LR image, the TSC is extracted from the HR image and compared with the TSC derived from the generated SR image. Our TSC loss functions minimize the L1 distance between these two values. However, as in [22] an overemphasis on several components that contribute minimally to the training process can decrease the training accuracy. Furthermore, the output TSC values of the SR and HR images typically contain an excess of low-frequency components, which have little significance.

To address this issue, the generated HR mask is used by multiplying the TSC loss with both the SR and HR TSC values, effectively disregarding the influence of low-frequency components. This ensures that the loss computation primarily considers discrepancies in the high-frequency regions. The formulated TSC loss is described as follows.

(4) $$L_{TSC}^{G}={E\|P^{HR}{}^{\circ}M_{h}^{HR}-P^{SR}{}^{\circ}M_{h}^{HR}\|}_{1},$$

where *P^HR^* and *P^SR^* are the TSC values of HR and SR images, respectively. By employing these methodologies, the previously mentioned issue is decreased and training efficiency is enhanced through the selective extraction of components in the high-frequency region.

### 2.3. Overall Network Architecture

**Figure 3:**
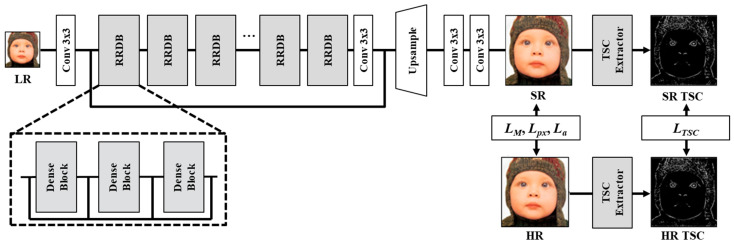
The overall network architecture of the proposed method includes the application of pixel-wise and adversarial losses, along with the introduced mutual and TSC losses. These losses are applied using the TSC extracted from both the SR and HR images through the TSC extractor during the training.

To reconstruct the SR output image, the SR decoder, introduced in ESRGAN [12], is used, incorporating residual in residual dense blocks (RRDBs). Additionally, the proposed loss functions are combined, namely, the mutual loss and TSC loss, and a TSC extractor is employed to generate a binary segmentation mask. The network architecture is depicted in Figure 3. ESRGAN proposes 23 RRDBs, where each RRDB comprises three dense blocks. Each dense block consists of five convolutional layers with 3 × 3 filters and 64 channel features, accompanied by four activation layers. The chosen scaling factor for this study is 4, and for the upscaling block, the sub-pixel convolutional layer proposed in [23] is used. The comprehensive loss function for training our generator is formulated as follows.

(5) $$L\left(G\right)=L_{M}^{G}+\gamma \cdot L_{Px}^{G}+\delta \cdot L_{a}^{G}+\eta \cdot L_{TSC}^{G},$$

where *γ*, *δ* and *η* denote the parameters of the different loss functions. The discriminator network in our proposed model follows the architecture used in ESRGAN [12], featuring two input channels and one output channel. However, to facilitate the application of the TSC loss involving the TSC value of the generated SR and HR images in our model, the image and the TSC are concatenated as follows.

(6) $$X^{SR}=Concat\left\{I^{SR},P^{SR}\right\},\;X^{HR}=Concat\left\{I^{HR},P^{HR}\right\},$$

where *X* represents our modified input for the discriminator with four input channels. The comprehensive loss function for optimizing the discriminator is expressed as follows.

(7) $$L\left(D\right)=-E\left[\log1-D(X^{SR})\right]-E\left[\log D(X^{HR})\right].$$

As a result, the proposed discriminator is equipped with four input channels and two output channels, enabling it to concurrently discriminate between the real image and the real TSC value, as well as the fake image and the fake TSC value, respectively.

## 3. Simulation Results

### 3.1. Implementation

The DIV2K dataset [24] was used to train our model, focusing on super-resolution performance. To assess our model’s effectiveness, evaluations were conducted on five widely recognized benchmarks: Set5 [25], Set14 [26], BSD100 [27], Urban100 [28], and General100 [5]. To obtain downsampled images, the MATLAB function was used, specifically considering a scaling factor of 4 in our experiments to ensure a fair comparison with other GAN-based SISR models. For evaluation metrics, the peak signal-to-noise ratio (PSNR) and structural similarity index (SSIM) were used, commonly used in SR performance assessments. Additionally, learned perceptual image patch similarity (LPIPS) was used to evaluate the perceptual quality of our images. PSNR is a metric to measure the quality of reconstructed or compressed images compared to the original image. The higher the PSNR value, the better the quality of the compressed or reconstructed image. The PSNR is calculated using the mean squared error (MSE) between the original and compressed images. The formula is as follows.

(8) $$PSNR=10\log_{10}\left(\frac{MAX^{2}}{MSE}\right),$$

where MAX is the maximum possible pixel value of the image. MSE is calculated as follows.

(9) $$MSE=\frac{1}{mn}\sum_{i=0}^{m-1}\sum_{j=0}^{n-1}{\left[I\left(i,j\right)-K\left(i,j\right)\right]}^{2},$$

where $I\left(i,\;j\right)$ is the pixel value at position $\left(i,\;j\right)$ in the original image, $K\left(i,\;j\right)$ is the pixel value at position $\left(i,\;j\right)$ in the compressed image, and m and n are the width and height of the image. SSIM is a perceptual metric that quantifies the similarity between two images by considering changes in structural information, luminance, and contrast. SSIM is designed to improve upon simpler metrics like MSE or PSNR, which do not always align with human visual perception. The SSIM between two images x and y is defined as follows.

(10) $$SSIM\left(x,\;y\right)=\frac{\left(2\mu_{x}\mu_{y}+C_{1}\right)\left(2\sigma_{xy}+C_{2}\right)}{\left(\mu_{x}^{2}+\mu_{y}^{2}+C_{1}\right)\left(\sigma_{x}^{2}+\sigma_{y}^{2}+C_{2}\right)}\;,$$

where $\mu_{x}$ and $\mu_{y}$ are the means of images x and y. $\sigma_{x}^{2}$ and $\sigma_{y}^{2}$ are the variances of images x and y. $\sigma_{xy}$ is the covariance between the two images and $C_{1}$ and $C_{2}$ are small constants added to stabilize the division. LPIPS is a perceptual metric that compares two images based on feature representations extracted from neural networks. Unlike metrics like PSNR or SSIM, which operate directly on pixel values, LPIPS leverages deep network activations to assess visual similarity more aligned with human perception. Given two images x and y, the LPIPS score is computed as follows.

(11) $$LPIPS\left(x,\;y\right)=\sum_{l}d(f_{l}\left(x\right),\;f_{l}\left(y\right)),$$

where l represents different layers of a deep network. $f_{l}\left(x\right)$ and $f_{l}\left(y\right)$ are the feature representations of images x and y at layer l. $d\left(\cdot ,\cdot \right)$ is a distance function, typically the L2 distance or cosine similarity. The implementation details of the proposed method are as follows.

**Training Process and Hyperparameters:** To ensure a diverse and representative training process, random patches of size 32 × 32 pixels were extracted from the input low-resolution (LR) images. These patches were used to form input mini-batches, with each batch containing eight patches. The corresponding high-resolution (HR) patches, generated by the network, were of size 128 × 128 pixels and constituted our training data. For a fair comparison, the generator and discriminator architecture from ESRGAN [12] were used.
**Pre-Training with L1 Loss:** The training was initiated by pre-training the network using the L1 loss function for a total of 200 epochs. This pre-training phase allowed the network to learn the basic mappings between LR and HR image patches before introducing more complex loss functions.
**Proposed Loss and GAN Training:** After pre-training, our proposed loss functions were applied, and the generative adversarial network (GAN) loss was introduced in the subsequent 60 epochs, refining the network’s ability to produce realistic HR images.
**Perceptual and Contextual Loss Computation:** For the calculation of perceptual and contextual losses, a pre-trained VGG-19 network was used. The perceptual loss was computed by extracting features from the ReLU 5–4 layer of the VGG-19 model, while the contextual loss was based on features extracted from the ReLU 3–4 layer. These feature distances provided a high-level measure of similarity between the generated images and the ground truth HR images.
**Optimization Strategy and Hyperparameter Settings:** The Adam optimizer was employed for the training process, with hyperparameters set as follows: *β*_1_ = 0.9, *β*_2_ = 0.999, and *ϵ* = 1 × 10^−8^. Initially, the learning rate was set to 1 × 10^−4^, and to ensure gradual learning, it was halved at regular intervals, specifically, at epochs 10, 20, 30, 40, and 50. Several key hyperparameters were defined in the training process: The values of *α* and *β* in (3) were set to 0.5, and in (5), *γ*, *δ* and *η* were set to 0.01, 0.001, and 1, respectively. Additionally, *th_h_* and *th_l_* were used to generate the HR mask and were 0.1 and 0.08, respectively.

### 3.2. Results

To assess the efficacy of our proposed method through quantitative measures, a comparative analysis was conducted with established SISR techniques, including bicubic interpolation, SRGAN [8], EnhanceNet [11], ESRGAN [12], RCAN [21], and NatSR [29]. As in Table 1, our method achieved a significantly superior LPIPS score compared to other approaches. Simultaneously, our method demonstrates competitive PSNR and SSIM values. While RCAN and NatSR exhibit higher PSNR and SSIM values, the LPIPS metric, evaluating perceptual image quality, favors our proposed network. Notably, in comparison with other perceptual-driven SISR methods such as SRGAN and EnhanceNet, our approach outperforms them. Specifically, in contrast to ESRGAN, which shares the same network architecture as our model, our results underscore the effectiveness of our proposed approach in enhancing perceptual SISR performance.

**Figure 4:**
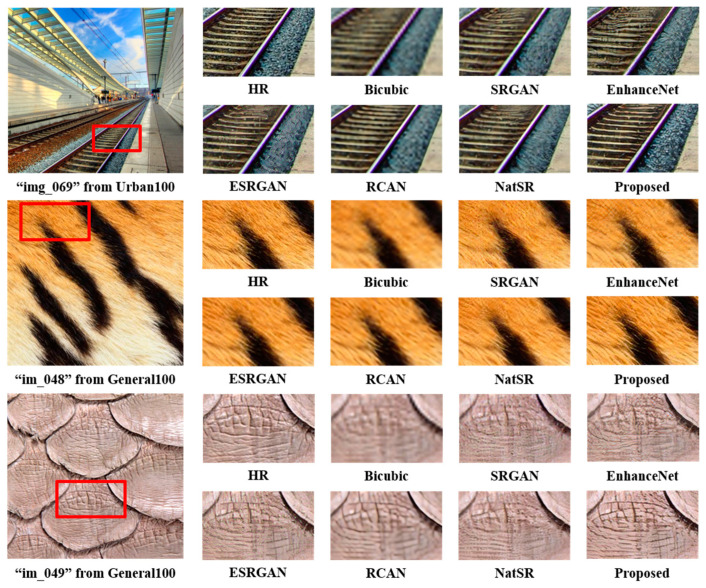
Visual comparison with other SISR methods for a scaling factor of 4 reveals that our approach produces more photorealistic images compared to alternative SISR methods.

For the visual assessment, a comparative analysis is conducted, visually contrasting our results with bicubic interpolation and the previously mentioned SISR models. As shown in Figure 4, our outcomes show a more photorealistic quality compared to other models. In the case of ‘img_069’ from Urban100, our method successfully restored rail boundaries without introducing blur or noise artifacts, a contrast to the images generated by alternative models. Similarly, for ‘im_048’ from General100, our generated SR image displayed a notable restoration of texture components, closely resembling the HR image. These observations extend to other images, where alternative methods struggle to restore intricate details. The qualitative results substantiate that our proposed method excels in restoring HR-like images with minimal distortions or blur artifacts.

**Figure 5:**
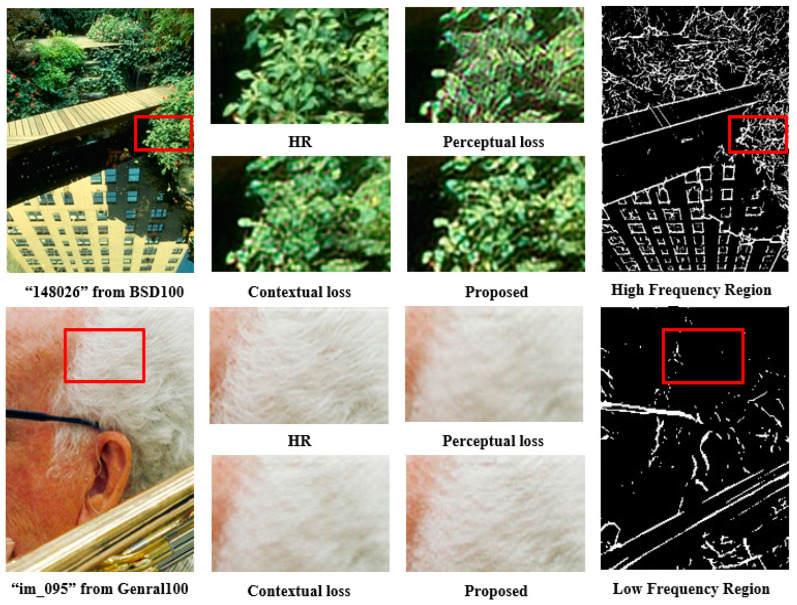
Performance comparison of the proposed mutual loss with perceptual and contextual losses when the same network and each loss function is individually used. To conduct a thorough evaluation, both high- and low-frequency regions are evaluated. In both regions, our method consistently demonstrates superior restoration, producing visually high-quality images compared to other methods.

**Effectiveness of Mutual Loss:** To validate the efficacy of the mutual loss, both perceptual and contextual losses separately were used into the same network as our method for an equitable comparison, keeping other loss functions constant. As illustrated in Figure 5 and detailed in Table 2, the utilization of the proposed mutual loss consistently outperforms methods employing only perceptual or contextual losses. In Figure 5, for the first image, particularly in regions with high-frequency components, the SR image produced by our proposed method appears more realistic compared to the output derived solely from perceptual and contextual losses. In the case of the second image with low-frequency components, our model generates more realistic details without introducing blur, resulting in an SR image that closely resembles HR quality. Furthermore, our method exhibits the lowest LPIPS score, indicating superior perceptual SISR performance, while maintaining competitive PSNR and SSIM values. These results affirm that our mutual loss facilitates the network in learning a reconstruction method suitable for both high-frequency and low-frequency regions, significantly contributing to the generation of perceptually high-quality SR images.

**Figure 6:**
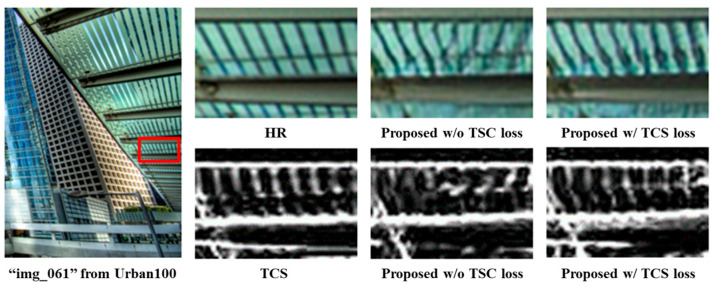
Visualization of the generated SR image and TSC to verify the effects of the TSC loss. Examination of the generated image and TSC reveals the beneficial impact of the proposed TSC loss in restoring the TSC of the SR image without distortions, particularly in regions containing high-frequency components.

**Effectiveness of Two-dimensional Structure Consistency Loss:** To evaluate the effectiveness of our TSC loss, Figure 6 compares the model output with and without the TSC loss, as depicted in Figure 6. Examining the HR image, the TSC of ‘img_061’ from Urban100 is clearly discernible. However, in the output of the model lacking the TSC loss, the TSC is not properly reconstructed. In contrast, the model incorporating the TSC loss exhibits superior reconstruction of the TSC, indicating improved performance in generating the TSC of the SR image. This visual comparison underscores the enhanced capability of our model in capturing perceptual-contextual information. Moreover, the impact of the TSC loss was quantitatively validated by comparing the model with the TSC loss against the model without the TSC loss, as detailed in Table 2. To validate the effectiveness of our TSC loss, the output of the model was visualized with the TSC loss and the output of the model without the TSC loss as shown in Figure 6. From the HR image, the TSC of the ‘img_061’ from Urban100 were clear. However, it is not properly reconstructed in the output of the model without the TSC loss, while better reconstructed in the model with the TSC loss, thus showing a better performance in reconstructing the TSC of the SR image than the model without the TSC loss. In addition, the effects of the TSC loss were quantitatively verified by comparing it with the model without the TSC loss in Table 2.

**Figure 7:**
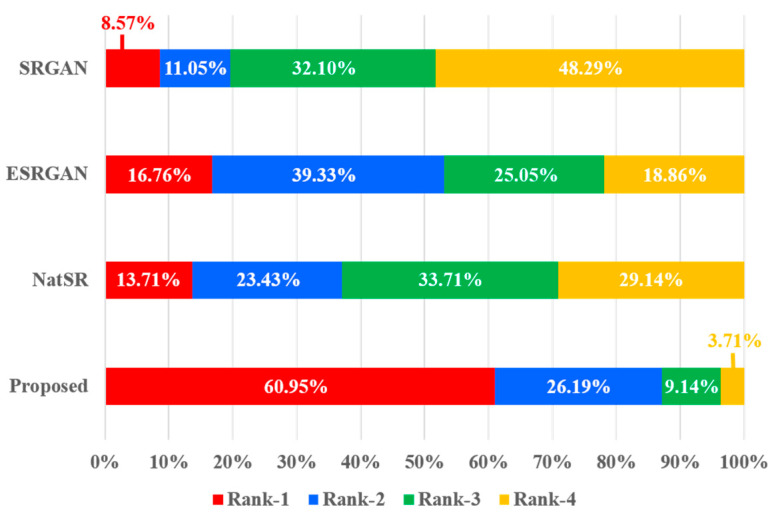
User study results between the proposed method and other perceptual-driven SISR methods (SRGAN [8], ESRGAN [12], and NatSR [29]). The proposed method obtains the most superior results among other SISR methods.

**User Study:** A user study was performed to compare the perceptual SR performance of the proposed method with other perceptual-driven SISR methods such as SRGAN [8], ESRGAN [12] and NatSR [29] using benchmark datasets. A total of 42 people participated in anonymous voting to evaluate randomly classified 25 images from rank 1 to 4. Figure 7 shows our summarized results. Each rank has a total of 1050 votes, where proposed method obtained 640 votes and 275 votes in rank 1 and rank 2, respectively. Excepting the proposed method, ESRGAN showed the highest performance, obtaining 176 votes and 413 votes in rank 1 and rank 2; meanwhile, NatSR and SRGAN failed to achieve satisfactory results. Consequently, the comparison result verified that proposed method outperforms other perceptual-driven SISR methods in generating more visually clear images to the human raters.

## 4. Conclusions

In this paper, our approach leveraged adaptive frequency region analysis using two-dimensional structure consistency (TSC), effectively addressing challenges related to the restoration of missing high-frequency components and enhancing the generation of image details in low-frequency regions. The application of the proposed mutual loss further boosted perceptual SISR performance by dynamically adjusting the training methodology for each frequency region based on the generated TSC-derived HR mask. Moreover, a novel allocation of the TSC loss was introduced to the high-frequency region, facilitating the reconstruction of high-frequency components in the SR image without introducing distortions. In both quantitative and qualitative experiments, our method attained the highest perceptual SR performance compared to state-of-the-art SISR models. These findings underscored the effectiveness of frequency domain analysis and adaptive training method application in enhancing the perceptual quality of SR images.

However, the proposed method fails to precisely analyze high-frequency components and, as a result, faces difficulties in accurately analyzing local features for resolution enhancement. Therefore, the future work will focus on refining methods to extract more accurate high-frequency components. Additionally, as high-frequency regions encompass various components such as edges, textures, and line segments, the next plan is to explore more efficient restoration methods tailored to each high-frequency component.

## Figures and Tables

**Table 1:** Performance comparison with state-of-the-art perceptual-driven SISR methods on commonly used benchmark datasets for scaling factor 4. The proposed method demonstrates superior perceptual SR performance, as verified by LPIPS, and comparable PSNR and SSIM values, simultaneously.

Method	Metric	Set5	Set14	BSD100	Urban100	General100
	PSNR	28.420	25.990	25.956	23.137	28.006
Bicubic	SSIM LPIPS	0.8245 0.3407	0.7837 0.4384	0.6672 0.5240	0.9008 0.4726	0.8278 0.4384
	PSNR	29.833	26.743	25.881	24.419	29.281
SRGAN [8]	SSIM LPIPS	0.8567 0.1102	0.7861 0.2205	0.6420 0.2551	0.9314 0.2088	0.8483 0.1438
	PSNR	28.838	25.932	25.255	23.623	28.331
EnhanceNet [11]	SSIM LPIPS	0.8409 0.1205	0.7762 0.1648	0.6351 0.2081	0.9317 0.1701	0.8343 0.1366
	PSNR	30.604	27.352	25.948	25.317	30.352
ESRGAN [12]	SSIM LPIPS	0.8801 0.0980	0.8092 0.1673	0.6648 0.1978	0.9511 0.1476	0.8729 0.1110
	PSNR	32.708	28.819	27.836	27.088	32.038
RCAN [21]	SSIM LPIPS	0.9125 0.1705	0.8564 0.2759	0.7446 0.3598	0.9695 0.19912	0.9033 0.1674
	PSNR	30.981	27.418	26.443	25.460	30.342
NatSR [29]	SSIM LPIPS	0.8796 0.0943	0.8120 0.1765	0.6826 0.2115	0.9503 0.1502	0.8718 0.1117
	PSNR	30.806	27.200	25.817	25.250	30.139
Proposed	SSIM LPIPS	0.8774 0.0661	0.8062 0.1314	0.6683 0.1671	0.9528 0.1250	0.8685 0.0890

**Table 2:** Performance comparison of methods with different components for scaling factor 4. The best performance is highlighted in red, while the second-best one is highlighted in blue (PSNR ↑/SSIM ↑/LPIPS ↓).

Dataset	Ours Without PC Loss	Ours with Contextual Loss	Ours with Perceptual Loss	Ours with Mutual Loss
Set5	30.632/0.8728/0.0702	31.336/0.8880/0.0824	30.804/0.8884/0.0984	30.806/0.8774/0.0661
Set14	26.750/0.7982/0.1328	27.465/0.8160/0.1462	27.422/0.8072/0.1673	27.200/0.8062/0.1314
BSD100	25.599/0.6642/0.1918	26.421/0.6839/0.1803	25.948/0.6648/0.1978	25.817/0.6683/0.1671
Urban100	25.130/0.9515/0.1266	25.571/0.9561/0.1321	25.317/0.9511/0.1476	25.250/0.95280.1250
General100	29.772/0.8611/0.0921	30.581/0.8762/0.0959	30.432/0.8721/0.1023	30.139/0.8685/0.0890

## Data Availability

Dataset available on request from the authors.
